# Temporal evolution of electrographic seizures in newborn infants with hypoxic-ischaemic encephalopathy requiring therapeutic hypothermia: a secondary analysis of the ANSeR studies

**DOI:** 10.1016/S2352-4642(23)00296-1

**Published:** 2024-03

**Authors:** Andreea M Pavel, Janet M Rennie, Linda S de Vries, Sean R Mathieson, Vicki Livingstone, Mikael Finder, Adrienne Foran, Divyen K Shah, Ronit M Pressler, Lauren C Weeke, Eugene M Dempsey, Deirdre M Murray, Geraldine B Boylan, ANSeR Consortium, Elena Pavlidis, Liudmila Kharoshankaya, Liam Marnane, Gordon Lightbody, Jackie O'Leary, Mairead Murray, Jean Conway, Denis Dwyer, Andrey Temko, Taragh Kiely, Anthony C Ryan, Subhabrata Mitra, Mona C Toet, Mats Blennow, Ingela Edqvist, Raga M Pinnamaneni, Jessica Colby-Milley, Nicola Openshaw-Lawrence, Olga Kapellou, Alexander C van Huffelen

**Affiliations:** a INFANT Research Centre and Department of Paediatrics and Child Health, University College Cork, Cork, Ireland; b Institute for Women's Health, University College London, London, UK; c Utrecht Brain Center, University Medical Center Utrecht, Utrecht University, Utrecht, Netherlands; d Department of Neonatal Medicine, Karolinska University Hospital, Stockholm, Sweden; e Division of Paediatrics, Department CLINTEC, Karolinska Institutet, Stockholm, Sweden; f Department of Neonatal Medicine, Rotunda Hospital, Dublin, Ireland; g Royal London Hospital, London, UK; h London School of Medicine and Dentistry, Queen Mary University of London, London, UK; i Department of Clinical Neurophysiology, Great Ormond Street Hospital for Children NHS Trust, London, UK

## Abstract

**Background:**

Despite extensive research on neonatal hypoxic-ischaemic encephalopathy, detailed information about electrographic seizures during active cooling and rewarming of therapeutic hypothermia is sparse. We aimed to describe temporal evolution of seizures and determine whether there is a correlation of seizure evolution with 2-year outcome.

**Methods:**

This secondary analysis included newborn infants recruited from eight European tertiary neonatal intensive care units for two multicentre studies (a randomised controlled trial [NCT02431780] and an observational study [NCT02160171]). Infants were born at 36^+0^ weeks of gestation with moderate or severe hypoxic-ischaemic encephalopathy and underwent therapeutic hypothermia with prolonged conventional video-electroencephalography (EEG) monitoring for 10 h or longer from the start of rewarming. Seizure burden characteristics were calculated based on electrographic seizures annotations: hourly seizure burden (minutes of seizures within an hour) and total seizure burden (minutes of seizures within the entire recording). We categorised infants into those with electrographic seizures during active cooling only, those with electrographic seizures during cooling and rewarming, and those without seizures. Neurodevelopmental outcomes were determined using the Bayley's Scales of Infant and Toddler Development, Third Edition (BSID-III), the Griffiths Mental Development Scales (GMDS), or neurological assessment. An abnormal outcome was defined as death or neurodisability at 2 years. Neurodisability was defined as a composite score of 85 or less on any subscales for BSID-III, a total score of 87 or less for GMDS, or a diagnosis of cerebral palsy (dyskinetic cerebral palsy, spastic quadriplegia, or mixed motor impairment) or epilepsy.

**Findings:**

Of 263 infants recruited between Jan 1, 2011, and Feb 7, 2017, we included 129 infants: 65 had electrographic seizures (43 during active cooling only and 22 during and after active cooling) and 64 had no seizures. Compared with infants with seizures during active cooling only, those with seizures during and after active cooling had a longer seizure period (median 12 h [IQR 3–28] *vs* 68 h [35–86], p<0·0001), more seizures (median 12 [IQR 5–36] *vs* 94 [24–134], p<0·0001), and higher total seizure burden (median 69 min [IQR 22–104] *vs* 167 min [54–275], p=0·0033). Hourly seizure burden peaked at about 20–24 h in both groups, and infants with seizures during and after active cooling had a secondary peak at 85 h of age. When combined, worse EEG background (major abnormalities and inactive background) at 12 h and 24 h were associated with the seizure group: compared with infants with a better EEG background (normal, mild, or moderate abnormalities), infants with a worse EEG background were more likely to have seizures after cooling at 12 h (13 [54%] of 24 *vs* four [14%] of 28; odds ratio 7·09 [95% CI 1·88–26·77], p=0·0039) and 24 h (14 [56%] of 25 *vs* seven [18%] of 38; 5·64 [1·81–17·60], p=0·0029). There was a significant relationship between EEG grade at 12 h (four categories) and seizure group (p=0·020). High total seizure burden was associated with increased odds of an abnormal outcome at 2 years of age (odds ratio 1·007 [95% CI 1·000–1·014], p=0·046), with a medium negative correlation between total seizure burden and BSID-III cognitive score (*r*_S_=–0·477, p=0·014, n=26).

**Interpretation:**

Overall, half of infants with hypoxic-ischaemic encephalopathy had electrographic seizures and a third of those infants had seizures beyond active cooling, with worse outcomes. These results raise the importance of prolonged EEG monitoring of newborn infants with hypoxic-ischaemic encephalopathy not only during active cooling but throughout the rewarming phase and even longer when seizures are detected.

**Funding:**

Wellcome Trust, Science Foundation Ireland, and the Irish Health Research Board.

**Research in context**

**Evidence before this study**

Detailed information of seizure evolution in neonatal hypoxic-ischaemic encephalopathy during therapeutic hypothermia with a focus on the rewarming phase is sparse. We did a MEDLINE literature search for randomised controlled trials and observational studies published in English up to Dec 20, 2021, using the search terms “neonatal encephalopathy”, “therapeutic hypothermia”, “seizures after cooling”, and “seizures during rewarming”. We found only two studies that investigated the persistence of seizures beyond active cooling and long-term outcomes. One study (n=23) reported that 60% of infants had seizures during rewarming and found that recurrent seizures within 24 h of rewarming were associated with epilepsy at age 1 year. Another study (n=120) reported that 23% of infants had seizures during rewarming and found an association between seizures during rewarming and death and disability at age 2 years. All previous published studies either analysed small cohorts of infants with hypoxic-ischaemic encephalopathy or detected seizures using amplitude-integrated electroencephalography (EEG) monitoring, which is known to have limitations when used in newborn infants. Based on the increasing evidence from published studies that showed a correlation between high seizure burden and adverse outcomes in infants with hypoxic-ischaemic encephalopathy, independent of the background severity and antiseizure medication, it is paramount to investigate further the seizure evolution in hypoxic-ischaemic encephalopathy in the hope of developing targeted interventions for improved outcomes.

**Added value of this study**

To the best of our knowledge, this is the first study to report a comprehensive description of the burden and evolution of electrographic seizures using conventional EEG monitoring, the gold-standard monitoring recommended by international guidelines, in a large multicentre cohort of newborn infants with hypoxic-ischaemic encephalopathy throughout therapeutic hypothermia. Background patterns and seizures were annotated for the entire monitoring period, including the rewarming phase, by experts in neonatal EEG interpretation. We highlighted the different patterns of seizure evolution in this cohort (seizures during and beyond active cooling), with two seizure burden peaks during therapeutic hypothermia at approximately 24 h and 85 h of age. Compared with infants with seizures during active cooling only, infants with seizures during rewarming and beyond were more likely to have a worse EEG background (major abnormalities and inactive background) at 12 h and 24 h. Infants with seizures beyond active cooling had a significantly higher odds of an abnormal outcome than infants without seizures. These infants also had a significantly higher total seizure burden than infants with seizures only during cooling, and this was associated with abnormal outcome at 2 years of age.

**Implications of all the available evidence**

The current findings highlight the need for continuous EEG monitoring throughout cooling and rewarming to support effective management of newborns with hypoxic-ischaemic encephalopathy, as well as the need for targeted interventions to augment the effect of therapeutic hypothermia.

## Introduction

Despite improvements in antenatal care and fetal monitoring, perinatal hypoxic-ischaemic injury is the most common cause of neonatal encephalopathy.^1^, ^2^ Hypoxic-ischaemic encephalopathy is a worldwide public health problem leading to death and long-term adverse outcome, with an incidence of approximately 1·5–3·0 per 1000 livebirths in high-income countries.^3^, ^4^

Therapeutic hypothermia is currently the only treatment shown to be of benefit for infants with moderate and severe hypoxic-ischaemic encephalopathy in high-income countries.^5^, ^6^ However, despite the introduction of therapeutic hypothermia, studies report that up to a third of survivors have a high risk of lifelong debilitating sequelae, such as cerebral palsy, epilepsy, and other intellectual and behavioural impairments.^7^, ^8^

The reported incidence of seizures in hypoxic-ischaemic encephalopathy is 30–65% in studies published since the introduction of therapeutic hypothermia.^9^, ^10^ Several studies have described the evolution of seizures in infants with hypoxic-ischaemic encephalopathy during therapeutic hypothermia,^11^, ^12^ but very few focused on the seizures after the active cooling phase (ie, rewarming).^13^, ^14^, ^15^, ^16^ Previous studies have shown a correlation between high seizure burden and death or long-term disability, or a combination of these outcomes, in infants with hypoxic-ischaemic encephalopathy, independent of the background severity and antiseizure medication.^17^, ^18^, ^19^, ^20^ The effect of seizures after the active cooling phase is not yet fully understood, but studies have suggested an association between seizures in the rewarming period and abnormal outcomes.^13^, ^14^ All previous published studies either analysed small numbers of infants with hypoxic-ischaemic encephalopathy or detected seizures by amplitude-integrated EEG monitoring, which is known to have limitations for the detection of all seizures in newborn infants.^21^ Thus, by pooling data from two large European multicentre cohorts of infants with hypoxic-ischaemic encephalopathy undergoing therapeutic hypothermia and prolonged conventional EEG monitoring, we aimed to carefully describe the true temporal evolution of electrographic seizures and to assess the relationship between seizure burden and long-term neurodevelopmental outcomes. Our hypothesis was that a significant number of infants with hypoxic-ischaemic encephalopathy undergoing therapeutic hypothermia will have a prolonged seizure period, with seizures persisting beyond the active cooling phase, which will have implications for the duration of EEG monitoring.

## Methods

### Study design and participants

This is a secondary analysis of data from newborn infants recruited as part of two multicentre studies (an observational study and a randomised controlled trial of the ANSeR Consortium) across eight European tertiary neonatal intensive care units. Newborn infants born after 36^+0^ weeks' gestational age requiring EEG monitoring for clinical reasons were eligible for inclusion in the main studies. The main results of the two studies have already been published,^22^, ^23^ and the study protocols are available on ClinicalTrials.gov (NCT02160171 and NCT02431780). Specifically, for this analysis, we selected and analysed all infants with a diagnosis of moderate or severe encephalopathy due to hypoxic ischaemia and undergoing therapeutic hypothermia. The diagnosis and severity of hypoxic-ischaemic encephalopathy were established by clinical teams involved in the care of the newborn infants at each participating centre, based on signs of perinatal asphyxia with clinical signs of moderate or severe encephalopathy (the highest Sarnat score within 24 h of life) and corroborated retrospectively with concomitant changes on the early EEG background pattern. Infants with a combined diagnosis (ie, sepsis, meningitis, or stroke), infants with mild hypoxic-ischaemic encephalopathy, and infants with hypoxic-ischaemic encephalopathy as a result of postnatal events were excluded from this analysis. The timing of EEG monitoring termination was decided by the clinical team at the participating site. Therefore, to assess the evolution of seizures in infants undergoing therapeutic hypothermia (including a rewarming phase of about 6–8 h and a minimum period of normothermia), infants with EEG monitoring discontinued at any point before 10 h from the start of rewarming were also excluded from this analysis.

Both studies had ethical approval at each participating centre by authorised ethics committees. Parental or guardian written informed consent was obtained for all participants.

### Procedures

The EEG recordings were performed using one of the following EEG machines: Nihon Kohden EEG (Neurofax EEG-1200; Tokyo, Japan), NicoletOne ICU Monitor (Natus, Orlando, FL, USA), or XLTek EEG (Natus). As recommended by international guidelines in neonatal EEG monitoring,^24^ we used the 10:20 EEG electrode modified neonatal system, placing disposable electrodes at F3, F4, C3, C4, Cz, T3, T4, O1/P3, O2/P4 positions, together with electrocardiography (ECG) and respiratory monitoring. Eight raw, bipolar EEG channels, as well as two aEEG signals (F3-C3 and F4-C4), and continuous video monitoring, were displayed continuously and available for interpretation by clinical teams as per local protocols.

All EEG recordings for all infants were included in this analysis. Electrographic seizures—defined as sudden, repetitive, and evolving stereotyped waveforms involving at least one EEG channel for at least 10 s^25^—were annotated twice by several certified neonatal neurophysiologists masked to the clinical background, but for this analysis we used a single consensus seizure annotation. Both electroclinical seizures and electrographic-only seizures were analysed in the same manner. Based on the seizure annotations, we calculated several seizure summary measures: seizure period (time between first and last electrographic seizure in hours), total seizure burden (duration of all seizures during the EEG monitoring in minutes), total number of seizures (all seizures during the EEG monitoring), maximum seizure burden (the maximum seizure burden within an hour [min/h]), status epilepticus (seizure burden of minimum 30 min within any given hour), and median seizure duration (the median duration of individual electrographic seizures in seconds). Seizure characteristics are derived from seizure burden and are used as descriptive parameters. Based on the fact that the outcome analysis was a secondary aim, we chose to use the most relevant seizure parameter, which is the total seizure burden.

A 1 h EEG epoch at age 12 h and 24 h was used to assess the EEG background activity as normal, mildly abnormal, moderately abnormal, major abnormalities, or inactive background, as previously described by our group.^26^ The background analysis was performed by a single neurophysiologist (SRM), who is an expert in neonatal EEG monitoring and masked to encephalopathy severity. These early timepoints were chosen because seizure onset in newborn infants with hypoxic-ischaemic encephalopathy is known to be within 24 h of birth and because the seizure treatment will further affect the EEG background pattern.^12^

For this study, infants were divided into three groups: infants with seizures exclusively up to the end of the active cooling period (no seizures beyond the start of rewarming); infants with seizures beyond the end of the active cooling period (during or beyond the rewarming period, with or without seizures before the end of active cooling); and infants without seizures throughout the EEG monitoring period.

### Outcomes

For the purpose of this analysis, the outcome was categorised as normal or abnormal, with abnormal outcome being defined as a combined outcome of death or disability at age 2 years. Neurodevelopmental outcomes were determined using the Bayley's Scales of Infant and Toddler Development, Third Edition (BSID‑III), the Griffiths Mental Development Scales (GMDS), or neurological assessment. BSID-III has three subscales—cognitive, language, and motor—and neurodevelopmental impairment (abnormal outcome) was defined as a composite score of 85 or below on any one of the three subscales. For GMDS, an abnormal outcome was defined as a total score of 87 or less. Neurological assessment was undertaken by a paediatric neurologist who assessed muscle tone, power, and deep tendon reflexes. The outcome was also considered to be abnormal if the infant had a formal diagnosis of cerebral palsy, including dyskinetic cerebral palsy, or spastic quadriplegia, mixed motor impairment, or epilepsy, or in case of death. A separate analysis was performed for infants with BSID-III scores.

### Statistical analysis

All tests were two sided, and a p value of less than 0·05 was considered to be significant. Continuous variables are summarised using the median (IQR), and categorical variables are summarised using frequency (percentage). We investigated differences between groups using the Mann Whitney *U* test or the Kruskal-Wallis test for continuous variables and the χ^2^ test or Fisher's exact test (in the case of small expected counts) for categorical variables. Due to the exploratory nature of this study and because reducing the probability of type I error would increase the probability of type II error, we did not adjust for multiple comparisons in the analysis.^27^ The effect size (*r*) for the Mann-Whitney *U* test was calculated using the formula:

$$r=\frac{z}{\sqrt{n}}$$

where *z* is the standardised test statistic and n is the number of infants included in the analysis. An effect size of 0·1 was considered to be small, 0·3 was considered to be moderate, 0·5 was considered to be large.^28^ For infants with seizures, we used binary logistic regression to assess the relationship between EEG background activity at age 12 h and seizure group (infants with seizure up to the end of the cooling phase=0 and infants with seizure after the cooling phase=1). Seizure group was the dependent variable and EEG background was the independent variable in the model. Odds ratios (ORs) and their corresponding 95% CIs are presented. To determine effect sizes, ORs were converted to Cohen's *d* using the formula:^29^

$$d=\frac{\ln(\text{OR})\times \sqrt{3}}{\pi }$$

A Cohen's *d* effect size of 0·2 was considered to be small, 0·5 was considered to be moderate, and 0·8 was considered to be large.^28^ All data used in the logistic regression model were used to calculate the area under a receiver operating characteristic curve (AUC). The AUC measures the predictive performance of the independent variables in the model and can range from 0·5 (discrimination no better than chance) to 1 (perfect discrimination). We also used logistic regression to assess relationships between neurodevelopmental outcome, seizure status, and total seizure burden. We assessed relationships between total seizure burden and BSID-III subscale scores using Spearman's correlation coefficient (*r*_S_). The seizure burden plots were generated as the mean value of each seizure burden plot for each baby, and then the mean is convolved with a normalised 10 h Hamming window to generate smooth plots. Statistical analysis was performed using IBM SPSS Statistics (version 28.0) and Stata (version 17.0) for the logistic regression and AUC analysis.

### Role of the funding source

The funders had no role in study design, data collection, data analysis, data interpretation, or writing of the report.

## Results

**Figure 1:**
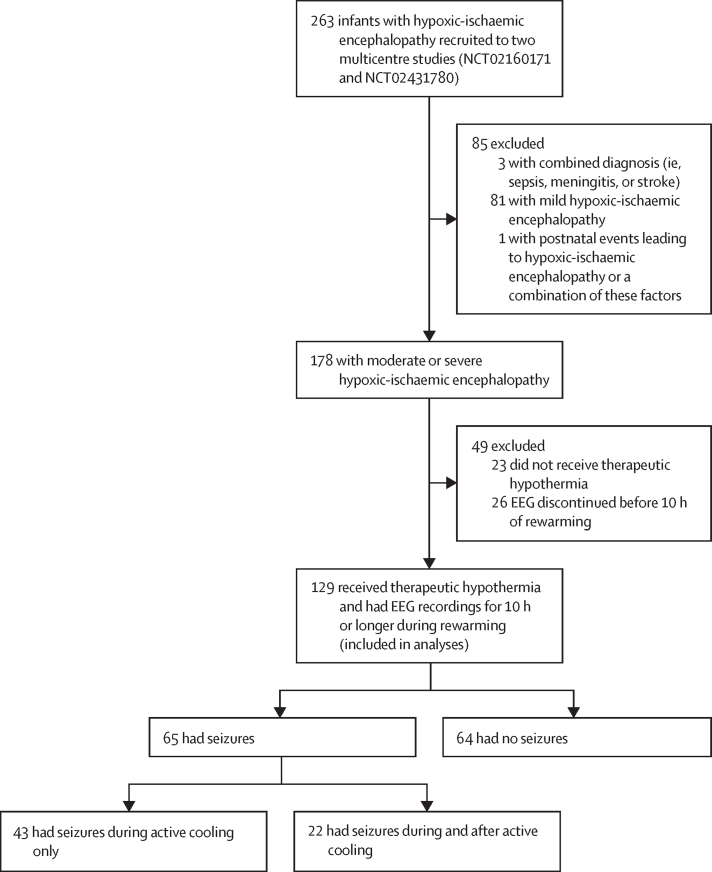
Study flow. EEG=electroencephalography.

Between Jan 1, 2011, and Feb 7, 2017, 263 infants with hypoxic-ischaemic encephalopathy were recruited to both studies. Of these infants, 178 (68%) had moderate or severe hypoxic-ischaemic encephalopathy. After excluding 23 infants who did not receive therapeutic hypothermia and 26 infants with EEG recordings for less than 10 h during rewarming, 129 infants were included in this analysis: 65 infants with electrographic seizures (43 with seizures during active cooling only and 22 with seizures after active cooling, with or without seizures during active cooling) and 64 without seizures (figure 1).

There were no significant differences between the study groups in terms of gestational age and birthweight, place and mode of birth, need for ventilation within 10 min of birth, cord pH, and age at start, and duration, of therapeutic hypothermia phases (table 1). Overall, 109 (84%) of 129 newborn infants included in the analysis had been actively cooled for 70–74 h (ten were cooled slightly more and ten were cooled less); however, there was no statistical difference in cooling duration between the study groups. There were significantly fewer male infants among those with seizure during active cooling only than among those without seizures, and Apgar scores at 1 min were significantly higher for infants without seizures than for those with seizures. Overall, EEG monitoring was started at median age of 7 h (IQR 4–13), and continued for a median duration of 93 h (85–104). EEG monitoring started significantly later for infants without seizures than for those with seizures during active cooling only. There was a significant difference between groups in clinical hypoxic-ischaemic encephalopathy severity (the majority of infants with seizures during active cooling only and those without seizures had moderate hypoxic-ischaemic encephalopathy, and the majority of infants with seizures during and after active cooling had severe hypoxic-ischaemic encephalopathy).

**Table 1:** Participant demographics, clinical characteristics, and EEG background patterns

		**Infants with seizures during active cooling only (n=43)**	**Infants with seizures during and after active cooling (n=22)**	**Infants with no seizures (n=64)**	p value*
Place of birth		..	..	..	0·16
	Born in recruiting hospital	25 (58%)	8 (36%)	27 (42%)	..
	Born outside recruiting hospital	18 (42%)	14 (64%)	37 (58%)	..
Gestational age, weeks		40·4 (39·6–41·3)	39·7 (39·0–40·6)	40·3 (39·5–41·0)	0·16
Mode of delivery		..	..	..	0·38
	Emergency (assisted vaginal delivery and emergency caesarean section)	25 (58%)	15 (68%)	44/62 (71%)	..
	Non-emergency (unassisted vaginal delivery and elective caesarean section)	18 (42%)	7 (32%)	18/62 (29%)	..
Birthweight, g		3580 (3110–3999)	3385 (3021–3694)	3523 (3082–4011)	0·35
Sex		..	..	..	0·034
	Male	21 (49%)	16 (73%)	46 (72%)	..
	Female	22 (51%)	6 (27%)	18 (28%)	..
	Pairwise p value	0·016†	..	..	..
Apgar score 1 min‡		1 (0–2)	1 (0–1)	2 (1–3)	0·0006
	Pairwise p value	..	..	0·0077§, 0·0006¶	..
Apgar score 5 min‡		3 (2–5)	3 (0–4)	4 (2–6)	0·047
	Pairwise p value	..	0·044†	..	..
Apgar score 10 min‖		5 (3–6)	4 (1–6)	5 (4–8)	0·055
Assisted ventilation within 10 min of birth		34/42 (81%)	20 (91%)	50 (78%)	0·42
Cord pH**		7·03 (6·81–7·22)	7·00 (6·80–7·16)	7·04 (6·88–7·21)	0·66
Age at start of cooling, h		2 (1–4)	2 (1–6)	3 (1–6)	0·23
Duration of cooling, h		72 (72–72)	72 (72–72)	72 (72–73)	0·32
Age at start of rewarming, h		75 (73–76)	73 (73–78)	75 (73–78)	0·20
Age at start of EEG monitoring, h		4 (3–11)	7 (4–12)	9 (5–17)	0·0007
	Pairwise p value	0·0002†	..	..	..
EEG monitoring duration, h		94 (86–103)	105 (90–137)	90 (75–100)	0·0044
	Pairwise p value	0·045†	0·0031†	..	..
EEG monitoring duration from after end of cooling, h		24 (17–35)	45 (28–66)	27 (19–33)	0·0003
	Pairwise p value	0·0001¶	0·0005†	..	..
Severity of hypoxic-ischaemic encephalopathy		..	..	..	<0·0001
	Moderate	29 (67%)	7 (32%)	55 (86%)	..
	Severe	14 (33%)	15 (68%)	9 (14%)	..
	Pairwise p value	0·022†, 0·0063¶	<0·0001†	..	..
EEG background activity at 12 h		..	..	..	0·0053
	Normal background	0/35 (0%)	0/17 (0%)	1/38 (3%)	..
	Mild abnormalities	8/35 (23%)	2/17 (12%)	14/38 (37%)	..
	Moderate abnormalities	16/35 (46%)	2/17 (12%)	16/38 (42%)	..
	Major abnormalities	4/35 (11%)	5/17 (29%)	4/38 (11%)	..
	Inactive background	7/35 (20%)	8/17 (47%)	3/38 (8%)	..
	Pairwise p value	0·020¶	0·0007†	..	..
EEG background activity at 24 h		..	..	..	<0·0001
	Normal background	1/42 (2%)	1/21 (5%)	0	..
	Mild abnormalities	15/42 (36%)	3/21 (14%)	24/50 (48%)	..
	Moderate abnormalities	15/42 (36%)	3/21 (14%)	22/50 (44%)	..
	Major abnormalities	5/42 (12%)	6/21 (29%)	1/50 (2%)	..
	Inactive background	6/42 (14%)	8/21 (38%)	3/50 (6%)	..
	Pairwise p value	0·022¶	<0·0001†	..	..

Data are median (IQR), n (%), or n/N (%). EEG=electroencephalography.

* p values of less than 0·05 were considered to be significant; for comparisons between the three groups, the Kruskal-Wallis test was used for continuous data and the χ^2^ test or Fisher's exact test for categorical data.

† Versus those with no seizures.

‡ Data were missing for two infants with seizures during active cooling only and one with no seizures.

§ Versus those with seizures during active cooling only.

¶ Versus those with seizures during and after active cooling.

‖ Data missing for six infants with seizures during active cooling only, one with seizures during and after active cooling, and four with no seizures.

** Data missing for eight infants with seizures during active cooling only, four with seizures during and after active cooling, and seven with no seizures

There were significant differences in EEG background at 12 h (p=0·0053) and 24 h (p<0·0001) between the three study groups (table 1). Mild and moderate EEG abnormalities were seen in the majority of infants with seizures during active cooling only (24 [69%] of 35 at 12 h and 30 [71%] of 42 at 24 h) and in those without seizures (30 [79%] of 38 at 12 h and 46 [92%] of 50 at 24 h), whereas major EEG abnormalities or an inactive background were seen in the majority of infants with seizures during and after active cooling (13 [76%] of 17 at 12 h and 14 [67%] of 21 at 24 h). Comparing the EEG background between infants with seizures and those without seizures, there were significant differences at both 12 h (p=0·031) and 24 h (p=0·0008).

Comparison of infants included in this analysis (n=129) and infants excluded for EEG monitoring of less than 10 h during rewarming phase (n=26) showed significant differences for cord pH (lower in the group of excluded infants) and duration of EEG monitoring (shorter monitoring in the group of excluded infants; appendix pp 4–6). There were no significant differences between the two groups for hypoxic-ischaemic encephalopathy severity, seizure occurrence, outcome availability, and presence of an abnormal outcome (appendix pp 4–6).

As expected, based on the study group definition, infants with seizures during and after active cooling had a longer seizure period, more seizures, and a higher total seizure burden than those with seizure during active cooling only (table 2). Infants with seizures during and after active cooling were significantly older when their first seizure occurred (p=0·0021) and when maximum seizure burden occurred (p<0·0001) than those with seizures during active cooling only. The same analysis was performed after excluding 24 infants with late EEG onset (after 16 hours of life), and no significant changes were noted.

**Table 2:** Seizure characteristics and EEG background activity

			**Infants with seizures during active cooling only (n=43)**	**Infants with seizures during and after active cooling (n=22)**	p value*
Seizure characteristics					
	Seizure period, h		12 (3–28)	68 (35–86)	<0·0001
	Total seizure burden, min		69 (22–104)	167 (54–275)	0·0033
		Seizure burden before end of cooling phase, min	69 (22–104)	103 (15–196)	0·25
		Seizure burden beyond cooling phase, min	..	44 (17–72)	..
	Total number of seizures		12 (5–36)	94 (24–134)	<0·0001
		Number of seizures before end of cooling phase	12 (5–36)	27 (9–81)	0·070
		Number of seizures beyond cooling phase	..	23 (8–52)	..
	Maximum seizure burden, min		22 (12–32)	23 (14–33)	0·59
		Hours after birth maximum seizure burden was reached	19 (13–28)	45 (26–80)	<0·0001
	Status epilepticus		13 (30%)	8 (36%)	0·62
	Age at first seizure, h		14 (9–18)	20 (15–50)	0·0021
	Median seizure duration, s		127 (78–455)	77 (63–119)	0·014
	Any antiseizure medication		38 (88%)	20 (91%)	1
	Number of antiseizure medication doses before EEG start		0 (0–1)	0 (0–1)	0·15
	Number of antiseizure medication doses after EEG start		1 (1–3)	3 (2–4)	0·033
EEG background activity at 12 h			..	..	0·020
	Normal background		0	0	..
	Mild abnormalities		8/35 (23%)	2/17 (12%)	..
	Moderate abnormalities		16/35 (46%)	2/17 (12%)	..
	Major abnormalities		4/35 (11%)	5/17 (29%)	..
	Inactive background		7/35 (20%)	8/17 (47%)	..
EEG background activity at 24 h			..	..	0·022
	Normal background		1/42 (2%)	1/21 (5%)	..
	Mild abnormalities		15/42 (36%)	3/21 (14%)	..
	Moderate abnormalities		15/42 (36%)	3/21 (14%)	..
	Major abnormalities		5/42 (12%)	6/21 (29%)	..
	Inactive background		6/42 (14%)	8/21 (38%)	..

Data are median (IQR), n (%), or n/N (%) unless otherwise stated. EEG=electroencephalograhy.

* The Mann Whitney *U* test was used for continuous data and the χ^2^ test or Fisher's exact test for categorical data.

When combined, worse EEG background (major abnormalities and inactive background) at 12 h and 24 h were associated with seizure group: compared with infants with a better EEG background (normal, mild, or moderate abnormalities), infants with a worse EEG background were more likely to have seizures after cooling at 12 h (13 [54%] of 24 *vs* four [14%] of 28; OR 7·09 [95% CI 1·88–26·77], p=0·0039) and 24 h (14 [56%] of 25 *vs* seven [18%] of 38; 5·64 [1·81–17·60], p=0·0029). There was a significant relationship between EEG grade at 12 h (four categories) and seizure group (p=0·020): infants with major abnormalities or an inactive background had significantly higher odds of having seizures after cooling than those with a moderate abnormality. The effect sizes were large (Cohen's *d*=1·27 for major abnormalities and *d*=1·22 for inactive background). Seizures during and after active cooling were more prevalent among infants with major EEG abnormalities at 12 h than among those with moderate EEG abnormalities at 12 h (OR 10·00 [95% CI 1·39–71·86], p=0·022). Similarly, seizures during and after active cooling were more prevalent among infants with inactive EEG at 12 h than among those with moderate EEG abnormalities at 12 h (9·14 [1·53–54·54], p=0·015). The ability of the EEG grade at 12 h of age to predict the study group (seizures during active cooling only *vs* seizures during and after active cooling) was high (AUC=0·741 [95% CI 0·603–0·880]).

**Figure 2:**
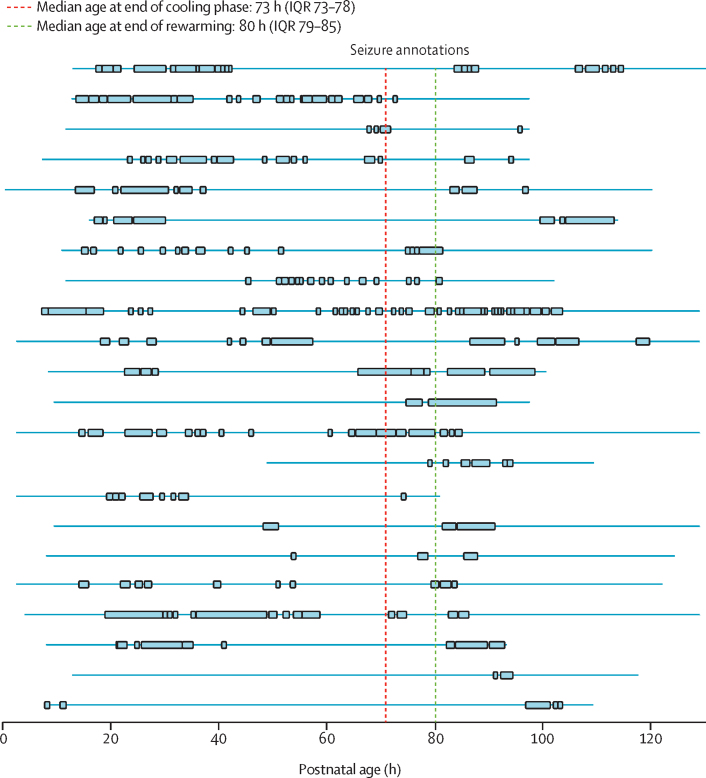
Individual temporal evolution of seizures in infants with electrographic seizures beyond the end of cooling phase (n=22) Each row corresponds to an infant and the order of infants is the same as the order of infants in the appendix (pp 6–7). The continuous thin blue line represents the entire EEG recording. The discontinuous thick blue lines represent seizures. EEG=electroencephalography.

Of the 22 infants with seizures during and after active cooling, 18 had seizures that started during active cooling; for three infants, seizures started only after the end of the active cooling phase (during rewarming period); and, for one infant (for whom therapeutic hypothermia and EEG monitoring were started late), seizures were recorded before the start of therapeutic hypothermia (at age 8 h; figure 2; appendix pp 6–7). 16 infants continued to have seizures after the rewarming process finished. Clinical details and individual hourly seizure evolution for infants with seizures during and after active cooling are presented in the appendix (pp 6–7).

**Figure 3:**
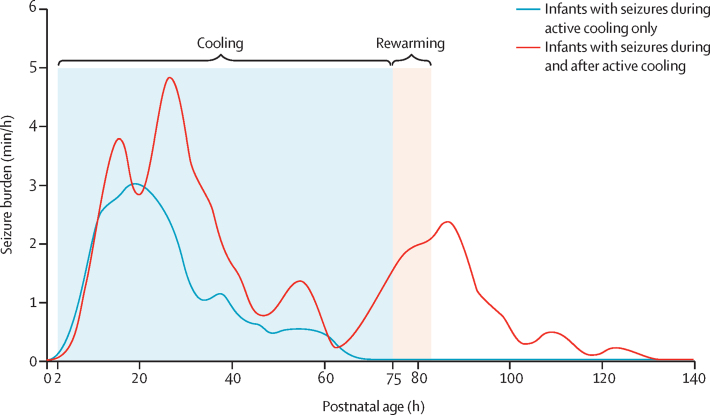
Seizure burden over the first 6 days by study group

In infants with seizures during active cooling only, the hourly seizure burden peaked at about age 20 h, and gradually decreased before the end of the cooling period (figure 3). Hourly seizure burden in infants with seizures during and after active cooling had two peaks: one at approximately 24 h and another smaller peak at approximately 85 h of age (figure 3).

Outcome data were available for 27 (63%) of 43 infants with seizures during active cooling only, 16 (73%) of 22 infants with seizures during and after active cooling, and 46 (72%) of 64 infants without seizures. Nine infants died (one with seizures during active cooling only, seven with seizures during and after active cooling, and one without seizures). In survivors, outcome was defined based on the BSID-III in 62 (78%) of 80 infants, or based on a detailed neurological assessment by a paediatric neurologist or a diagnosis of cerebral palsy, mixed motor impairment, or epilepsy in 17 (21%) of 80 infants (appendix p 8). The median age at assessment was 24 months (IQR 21–26). There were no significant differences in demographic characteristics between infants with outcome available and those without outcome available (data not shown).

Of the 89 infants with outcome data, 32 (36%) had an abnormal outcome of death or disability. An abnormal outcome occurred in ten (63%) of 16 infants with seizures during and after active cooling, nine (33%) of 27 infants with seizures during active cooling only, and 13 (28%) of 46 infants without seizures. There was a significant association between the study groups and outcome (p=0·046; pairwise comparison of infants with seizures during and after active cooling *vs* those without seizures, p=0·0090). There was also a significant association between the study groups and death (p<0·0001), although the sample size was small.

Overall, 19 (44%) of 43 infants who had seizures and 13 (28%) of 46 infants who did not have seizures had an abnormal outcome, but the effect size was small (Cohen's *d*=0·39) and the difference was not significant (OR 2·01 [95% CI 0·83–4·84], p=0·12). However, total seizure burden was significantly higher in infants with an abnormal outcome (median 28·1 min [IQR 0–183·4, n=32) than in infants with a normal outcome (0 min [0–50·9], n=57, p=0·022; small effect size: *r*=0·24). For every 1 min increase in total seizure burden, the odds of an abnormal outcome increased by 0·7% (OR 1·007 [95% CI 1·002–1·013], p=0·0074).

Restricting the analysis to infants with seizures, total seizure burden remained significantly higher for the 19 infants with an abnormal outcome (median 117·4 min [IQR 43·0–307·4]) than the 24 with a normal outcome (61·3 (19·6–99·9), p=0·024; moderate effect size: *r*=0·34). The relationship between total seizure burden and outcome remained significant after adjusting for length of EEG recording: OR 1·007 (95% CI 1·000–1·014), p=0·046.

A subgroup analysis was performed for infants with seizures and BSID-III scores available (appendix p 9). There was a significant medium negative correlation between total seizure burden and the BSID-III cognitive score (*r*_S_=–0·477, p=0·014, n=26), indicating that higher seizure burden was associated with worse cognition, and the correlations between total seizure burden and BSID‑III language score (*r*_S_=0·027, p=0·90, n=22) and total seizure burden and BSID-III motor score (*r*_S_=–0·389, p=0·066, n=23) were not significant.

## Discussion

This is one of the few studies to report a comprehensive description of the evolution of electrographic seizures using continuous multichannel EEG in a large multicentre cohort of newborn infants with hypoxic-ischaemic encephalopathy throughout cooling, rewarming, and beyond. Our results show that seizures evolve in different ways in infants undergoing therapeutic hypothermia. Infants with seizures during active cooling only (n=43) had a single seizure burden peak at approximately 20 h of age. Infants with seizures persisting beyond the active cooling phase (n=22) had a second seizure burden peak at approximately 85 h of age, and 16 of 22 infants continued to have seizures beyond the end of the rewarming phase, when they returned to a normothermic state. The temporal evolution of seizure burden is an important finding because it shows specific timepoints of high seizure burden during therapeutic hypothermia and beyond. This information could allow clinicians to anticipate the potential course of seizure burden evolution for infants with hypoxic-ischaemic encephalopathy, thus individualising EEG monitoring and review. More than half of infants with seizures during and after active cooling had an abnormal outcome, with death being the predominant outcome. Total seizure burden was significantly higher in newborn infants with an abnormal outcome. A high total seizure burden was also associated with lower BSID-III cognitive scores, but not with language and motor scores, at age 2 years. However, the sample size was probably too small to accurately assess this association. EEG background severity at age 12 h and 24 h was associated with seizure group. Compared with infants with seizures during active cooling only, infants with seizures during and after active cooling were more likely to have a worse early EEG background (major abnormalities and inactive background). Consistent with our findings, a recent study showed that persistent severely abnormal EEG background or late onset of seizures in hypoxic-ischaemic encephalopathy were associated with adverse outcome.^30^ This suggests the need for frequent reviews of EEG monitoring in the first 24 h and the need for prolonged EEG monitoring beyond the end of rewarming phase for infants with severely abnormal EEG background.

Death and long-term disability have also been reported by other studies in a significant proportion of infants with hypoxic-ischaemic encephalopathy, despite the introduction of therapeutic hypothermia, which underlines the need for other interventions.^31^ A high seizure burden has previously been linked to adverse outcomes despite encephalopathy severity and antiseizure medication, highlighting the need for rapid seizure detection and treatment in the management of newborns with hypoxic-ischaemic encephalopathy.^17^, ^18^, ^19^, ^20^ The persistence of seizures during rewarming and beyond is not fully understood and is understudied.^32^, ^33^, ^34^ The current study has shown different seizure evolution patterns in newborn infants with hypoxic-ischaemic encephalopathy that correlated with EEG background severity.

Two recently published studies used aEEG to examine the association between seizures during the rewarming phase of therapeutic hypothermia and long-term outcomes.^13^, ^14^ Chen and colleagues reported that 60% of infants had seizures on aEEG during rewarming and that recurrent seizures within 24 h of rewarming were associated with epilepsy at 1 year of age.^13^ Another study by Chalak and colleagues reported that 23% of infants had seizures during rewarming and found an association between seizures during rewarming and death and disability at 2 years of age.^14^ In our cohort, 34% of infants with seizures had electrographic seizures after the end of active cooling. A composite outcome of death and neurodisability at 2 years was available in 69% of infants in our cohort, with 63% mortality and morbidity in the group with seizures beyond active cooling. Consistent with previously mentioned studies, we found that a higher proportion of infants with seizures during and after active cooling in hypoxic-ischaemic encephalopathy had an abnormal outcome compared with infants with seizures during active cooling only or compared with infants without seizures, with a statistically significant difference only between infants with seizures during and after active cooling and those without seizures. It is difficult to demonstrate a clear relationship between seizure burden evolution and outcome (infants with seizures during active cooling only *vs* those with seizure during and after active cooling) because of other confounders, such as background hypoxic-ischaemic encephalopathy severity and the need for an increase in antiseizure medication. Infants with seizures during and after active cooling had a longer seizure period and a higher seizure burden, but also worse encephalopathy (higher clinical encephalopathy grade and worse EEG background activity) than those with seizures during active cooling only. As shown in the literature, encephalopathy severity, independently of seizure burden, is correlated with long-term outcomes. However, the medium negative correlation between total seizure burden and BSID-III cognitive scores supports the possible impact of seizure burden on long-term outcome, regardless of the timing of neonatal seizures.

As a result of methodological differences, it is difficult to directly compare our study with the study by Chalak and colleagues,^14^ but as shown on the hourly seizure burden plots, seizure burden was lower at the end of active cooling and increased again at the start of the rewarming phase, probably because of changes in metabolic demand with the increase in body temperature.^33^, ^35^ Of the 22 infants with seizures during and after active cooling, the first seizure was noted only during rewarming for three infants. However, for these infants, EEG monitoring started later at 8 h, 13 h, and 50 h of age, so seizures during the initial cooling period might have been missed. 16 infants with seizures during and after active cooling continued to have seizures after the end of the rewarming process. This is very concerning in terms of seizure detection, because most neonatal units would discontinue EEG monitoring shortly after rewarming and electrographic seizures would be missed. These findings are important and might have an important impact on the duration of EEG monitoring and long-term neurodevelopmental outcomes. Current guidelines recommend EEG monitoring during therapeutic hypothermia (cooling and rewarming) for newborn infants with hypoxic-ischaemic encephalopathy until normothermia is reached and until seizures are controlled.^24^ The current study clearly showed a late seizure burden peak at the end of therapeutic hypothermia in a significant proportion of infants with hypoxic-ischaemic encephalopathy, which shows the need for maintaining EEG monitoring beyond rewarming to capture rebound seizures, a well recognised phenomenon in animal studies.^36^

Some limitations have to be considered when interpreting the results. This was a retrospective analysis of a selected group of infants from a larger cohort of infants recruited for two studies powered to look at different outcomes. Although we showed an association between seizure burden and long-term outcome, the sample size was small and prospective studies powered to assess this relationship are necessary, which will also address other confounding factors, such as the impact of antiseizure medication (higher seizure burden is correlated with an increased need for antiseizure medication), brain injury evolution (worse brain injury is correlated with higher seizure burden), and socioeconomic factors. We noted no differences between the study groups in the need of early antiseizure medication; however, it is difficult to assess the impact of overall antiseizure medication on neurodevelopmental outcome without having a prospective standardised approach to seizure treatment. The absence of information on haemodynamic instability and cardiovascular support might be a contributing factor to seizure development and is another limitation that should be considered. Of 129 infants, 14 infants were older than 6 h when optimal cooling temperature was reached, defining the start of therapeutic hypothermia. Specific reasons for these delays were not captured in the original data collection; however, we know from clinical practice that such delays can happen when infants are outborn and that for some infants it takes longer to reach the target temperature. Additionally, when the infant is clinically unstable, therapeutic hypothermia might be delayed. In 24 newborn infants, EEG monitoring started later than 16 h after birth (cutoff chosen based on the median age at first seizure, which was 15·5 h). These infants were included in the analysis, with the potential risk of misclassifying infants as having no seizures if early seizures were missed (risk was acceptable due to likelihood of short seizure period, if any). However, the analysis was also performed excluding these infants with late EEG onset, and we found no significant changes in the results of the study. Some infants had EEG monitoring discontinued before the end of rewarming and were excluded from this analysis, reducing the cohort size. However, the demographics of included versus excluded infants were similar, as well as the age at start of EEG monitoring. Although the study cohort is considered to be large, outcome data were available for 89 (69%) of 129 infants, and not all infants had a standardised neurodevelopmental assessment. Although there was a clear difference in mortality before discharge between the study groups, due to the small sample size and because of all the above reasons, the outcome results should be interpreted with caution.

Despite these limitations, the current study has described in detail the seizure burden and seizure evolution in a large multicentre cohort of infants with hypoxic-ischaemic encephalopathy undergoing therapeutic hypothermia. All infants had prolonged continuous conventional EEG (most during the entire active cooling and rewarming periods), which is the gold-standard monitoring recommended by international guidelines.^24^ Background patterns and seizures were annotated for the entire monitoring period by experts in neonatal EEG interpretation. The current study also examined the relationship between seizure burden and EEG background patterns and long-term outcome.

A large proportion of infants with hypoxic-ischaemic encephalopathy undergoing therapeutic hypothermia continue to have seizures after the completion of active cooling, increasing the overall seizure burden, which might have an impact on long-term outcomes. Based on the results of our study, there is a clear need for continuous EEG monitoring during active cooling, rewarming, and beyond when seizures persist, especially in newborn infants with severely abnormal early EEG background patterns. However, prospective studies, powered to assess the relationship between the presence of seizures beyond cooling and long-term outcome in newborns with hypoxic-ischaemic encephalopathy, are required.

## Data sharing

For the current study, it is not possible to share the datasets. The clinical data were collected under a written proxy consent from the participants' guardians or parents, which did not include permission for sharing or open data. To be allowed to share this data under Irish Health Research Regulations we are required to re-consent families or to obtain approval by the Health Regulation Consent Declaration Committee.

## Declaration of interests

We declare no competing interests.
