# Supplementary material for: Temporal evolution of electrographic seizures in newborn infants with hypoxic-ischaemic encephalopathy requiring therapeutic hypothermia: a secondary analysis of the ANSeR studies
Source: Lancet Child Adolesc Health. 2024 Mar;8(3):214–24. doi: 10.1016/S2352-4642(23)00296-1 (PMC10864190; doi:10.1016/S2352-4642(23)00296-1)
Supplement: Supplementary appendix [file mmc1.pdf]

THE LANCET
Child & Adolescent Health

Supplementary appendix

This appendix formed part of the original submission and has been peer reviewed. We post it as supplied by the authors.

Supplement to: Pavel AM, Rennie JM, de Vries LS, et al. Temporal evolution of electrographic seizures in newborn infants with hypoxic-ischaemic encephalopathy requiring therapeutic hypothermia: a secondary analysis of the ANSeR studies. *Lancet Child Adolesc Health* 2024; published online Jan 18. https://doi.org/10.1016/S2352-4642(23)00296-1.

Contents

ANSeR Consortium 2

Table 1S. Comparison between infants included in the analysis (n=129) and infants excluded due to EEG monitoring for less than 10 hours of rewarming period (n=26)..4

Table 2S. Characteristics of infants in Group 2 (infants with electrographic seizures beyond the end of cooling phase) 6

Table 3S. Method of assessment on which outcome was based 8

Table 4S. BSID-III (The Bayley's Scales of Infant and Toddler Development - Third Edition) subscale scores (n=62)..9

ANSeR Consortium

- INFANT Research Centre and Department of Paediatrics and Child Health, University College Cork, Cork, Ireland

Andreea M Pavel, MD; *Eugene Dempsey, MD; Vicki Livingstone, PhD*; *Elena Pavlidis, MD; Liudmila Kharoshankaya, MD*; *Sean R Mathieson, PhD*; *Liam Marnane PhD*; *Gordon Lightbody PhD*; *Jackie O'Leary MSc*; *Mairead Murray MSc*; *Jean Conway MSc*; *Denis Dwyer BSc*; *Andrey Temko, PhD; Taragh Kiely, MSc; Anthony C Ryan, MD; Geraldine B Boylan PhD*

- Institute for Women's Health, University College London, London, UK

Janet M Rennie, MD; Subhabrata Mitra, PhD.

- Utrecht Brain Center, University Medical Center Utrecht, Utrecht University, The Netherlands

Linda S de Vries, PhD; *Lauren C Weeke, PhD; Mona C Toet, PhD;*

- Department of Neonatal Medicine and Division of Paediatrics, Department CLINTEC, Karolinska University Hospital, Karolinska Institutet, Stockholm, Sweden

Mikael Finder, PhD; *Mats Blennow PhD; Ingela Edqvist, RN*

- Rotunda Hospital, Dublin, Ireland

Adrienne Foran MD; *Raga Mallika Pinnamaneni, MD*; *Jessica Colby-Milley, MSc.*

- Royal London Hospital and Queen Mary University of London, London, UK

Divyen K Shah, MD; *Nicola Openshaw-Lawrence.*

- Department of Clinical Neurophysiology, Great Ormond Street Hospital for Children NHS Trust, London, UK

Ronit M Pressler, PhD.

- Homerton University Hospital NHS Foundation Trust, London, UK

Olga Kapellou, MD.

- Clinical Neurophysiology, University Medical Center Utrecht, Utrecht, The Netherlands *Alexander C van Huffelen, PhD.*

Table 1S. Comparison between infants included in the analysis (n=129) and infants excluded due to EEG monitoring for less than 10 hours of rewarming period (n=26)

	Included infants (n=129)	Excluded infants (n=26)	p-value$^{\alpha}$
Place of birth			0.45
Born in recruiting hospital	60 (47%)	10 (38%)	
Born outside recruiting hospital	69 (53%)	16 (62%)	
Gestational age, weeks	40 (39-41)	40(39-41)	0.62
Mode of delivery			0.94
Emergency (assisted vaginal delivery and emergency caesarean section)	84/127 (66%)	17 (65%)	
Non-emergency (unassisted vaginal delivery and elective caesarean section)	43/127 (34%)	9 (35%)	
Birth weight, g	3500(3084-3955)	3545(3237-4026)	0.27
Sex			0.63
Male	83 (64%)	18 (69%)	
Female	46 (36%)	8 (31%)	
Apgar score 1 minute$^{\Phi}$	1(0-3)	1(0-3)	0.82
Apgar score 5 minute$^{\Phi}$	4(2-5)	3(2-5)	0.68
Apgar score 10 minute$^{\$}$	5(3-7)	4(3-7)	0.44
Need of ventilation within 10 min of birth	104/128 (81)	17/25 (68)	0.14
Cord pH$^{\#}$	7.04(6.87-7.19)	6.88(6.79-7.07)	0.026
Age at start of cooling, hours	3(1-5)	3(2-5)	0.41
Duration of cooling, hours	72(72-73)	72(70-72)	0.046
Age at start of rewarming, hours	75(73-77)	75(72-77)	0.37
Age at start of EEG monitoring, hours	7(4-13)	9(5-18)	0.18
EEG monitoring duration, hours	93(85-104)	46(30-70)	<0.0001
HIE severity			0.20
Moderate	91 (71%)	15 (58%)	
Severe	38 (29%)	11 (42%)	

Seizures, yes	65 (50%)	10 (38%)	0.27
Outcome available, yes	95 (74%)	21 (81%)	0.45
Abnormal outcome	33/95 (35%)	11/21 (52%)	0.13

Data are median(IQR), n(%) or n/N (%). EEG – electroencephalogram; HIE – hypoxic-ischaemic encephalopathy.

[α]p values of less than 0.05 were considered to be significant; The Mann-Whitney U test was used for continuous data and the Chi-squared test for categorical data.

[Φ] Data were missing for 3 infants included in the study; [$] Data were missing for 11 infants included in the study and 1 infant excluded from the study; [#] Data were missing for 19 infants included in the study and 4 infants excluded from the study;

Table 2S. Characteristics of infants with seizures during and after active cooling

No	HIE grade	Gestational Age (weeks)	Sex	Assisted ventilation at 10 min	Cord pH	Age at start of cooling (hours)	Age at start of Rewarming (hours)	Age at start of EEG (hours)	EEG recording duration (hours)	Age at first seizure (hours)	Seizure period (hours)	Total seizure burden (min.)	Seizure number	Maximum seizure burden (min.)	Status epilepticus	Seizures			2 year Outcome
																During cooling phase	During rewarming phase	Post rewarming	
1	Severe	39	F	yes	-	1	73	12.7	209.3	17.71	97.04	307	103	32	yes	yes	yes	yes	Death
2	Moderate	38	M	yes	-	8	47	12.88	86.82	14.02	58.81	460	130	40	yes	yes	yes	yes	Abnormal
3	Severe	39	M	yes	6.85	6	78	11.71	89.42	67.92	28	6	5	1	no	yes	no	yes	Death
4	Moderate	40	M	yes	7.14	1	73	6.96	90.39	23.61	70.73	202	108	55	yes	yes	no	yes	Abnormal
5	Severe	41	F	yes	6.95	1	73	1.69	120.86	14.1	82.93	210	129	29	no	yes	yes	yes	Normal
6	Severe	41	M	yes	6.99	1	81	17.51	95.54	17.51	95.53	304	84	60	yes	yes	no	yes	Normal
7	Severe	39	F	yes	7.05	3	73	8.99	116.69	15.21	65.96	132	36	30	yes	yes	yes	no	-
8	Moderate	37	M	yes	7.34	3	75	10.48	94.74	45.59	35.59	59	22	7	no	yes	yes	no	Normal
9	Severe	41	M	yes	6.92	2	79	7.09	240.37	7.65	95.83	612	145	60	yes	yes	yes	yes	-
10	Severe	39	M	yes	7.31	1	74	3.21	134.46	18.6	113.3	265	213	30	yes	yes	no	yes	Death
11	Severe	39	M	no	7.18	5	77	6.99	93.14	23.06	75.32	364	210	34	yes	yes	yes	yes	Death
12	Severe	39	M	yes	6.69	4	73	8.12	90.07	75.2	15.89	117	213	17	no	no	yes	yes	Death
13	Severe	41	F	yes	7.01	1	73	3.65	151.9	14.56	70.31	212	104	27	no	yes	yes	no	Abnormal
14	Moderate	40	F	no	7.1	2	74	50.36	62.79	79.25	14.85	44	11	9	no	no	yes	yes	Normal
15	Severe	39	M	yes	6.8	1	48	4.11	72.51	19.79	54.53	33	21	7	no	yes	no	yes	Death
16	Moderate	37	M	yes	6.63	9	82	8.67	146.17	48.78	42.15	87	49	16	no	yes	yes	no	Death
17	Severe	39	M	yes	6.8	6	78	7.18	125.01	54	33.51	31	28	20	no	yes	yes	no	-

18	Severe	38	F	yes	-	1	73	2.49	120.8	14.6	69.26	112	30	22	no	yes	yes	no	Normal
19	Severe	40	M	yes	-	1	73	4.26	203.21	19.43	66.69	218	204	15	no	yes	yes	yes	-
20	Severe	39	M	yes	6.65	1	73	6.91	85.8	21.54	71.08	200	112	23	no	yes	yes	yes	-
21	Moderate	39	M	yes	7.18	9	81	13.43	106.55	91.36	2.75	2	4	1	no	no	yes	yes	-
22*	Moderate	40	M	yes	7.15	12	69	6.93	102.46	8.12	95.32	57	25	15	no	no	no	yes	Normal

HIE – hypoxic-ischaemic encephalopathy; M – male; F – female;

Infant 22* had seizures before start of therapeutic hypothermia. Order of infants in this table correspond with order of infants in Figure 2.

Table 3S. Method of assessment on which outcome was based

	Infants with seizures		Infants with no seizures (n=64)
	Infants with seizures during active cooling only (n=43)	Infants with seizures during and after active cooling (n=22)	
	n(%)	n(%)	n(%)
BSID-III	19 (44)	7 (32)	36 (56)
Paediatric/neurological assessment	7 (16)	1 (5)	9 (14)
GMDS	0	1 (5)	0
Death	1 (2)	7 (32)	1 (2)
Missing outcome	16 (37)	6 (27)	18 (28)

BSID-III – The Bayley's Scales of Infant and Toddler Development - Third Edition (Bayley 2006); GMDS – the Griffiths Mental Development Scales. Death was recorded during the neonatal period.

Table 4S. BSID-III (The Bayley's Scales of Infant and Toddler Development - Third Edition) subscale scores (n=62)

	Infants with seizures		Infants with no seizures (n=36)	
	Infants with seizures during active cooling only (n=19)	Infants with seizures during and after active cooling (n=7)		
	median(IQR)	median(IQR)	median(IQR)	p-value$^{\alpha}$
Cognitive	105 (95-110)	90 (70-110)	100 (90-110)	0.35
Language$^{\times}$	100 (93-109)	97 (90-103)	97 (86-109)	0.73
Motor$^{\&}$	100 (88-107)	94 (84-104)	103 (97-110)	0.23

$^{\alpha}$ from Kruskal-Wallis test

$^{\times}$n=17 in Infants with seizures during active cooling only, n=5 in Infants with seizures during and after active cooling and n=31 in Infants with no seizures

$^{\&}$ n=17 in Infants with seizures during active cooling only, n=6 in Infants with seizures during and after active cooling and n=27 in Infants with no seizures
